# Substance abuse and the risk of severe COVID-19: Mendelian randomization confirms the causal role of opioids but hints a negative causal effect for cannabinoids

**DOI:** 10.3389/fgene.2022.1070428

**Published:** 2022-12-13

**Authors:** M. Reza Jabalameli, Zhengdong D. Zhang

**Affiliations:** Department of Genetics, Albert Einstein College of Medicine, New York City, NY, United States

**Keywords:** Mendelian randomization, substance use disorder, GWAS, cannabinoids, inflammation

## Abstract

Since the start of the COVID-19 global pandemic, our understanding of the underlying disease mechanism and factors associated with the disease severity has dramatically increased. A recent study investigated the relationship between substance use disorders (SUD) and the risk of severe COVID-19 in the United States and concluded that the risk of hospitalization and death due to COVID-19 is directly correlated with substance abuse, including opioid use disorder (OUD) and cannabis use disorder (CUD). While we found this analysis fascinating, we believe this observation may be biased due to comorbidities (such as hypertension, diabetes, and cardiovascular disease) confounding the direct effect of SUD on severe COVID-19 illness. To answer this question, we sought to investigate the causal relationship between substance abuse and medication-taking history (as a proxy trait for comorbidities) with the risk of COVID-19 adverse outcomes. Our Mendelian randomization analysis confirms the causal relationship between OUD and severe COVID-19 illness but suggests an inverse causal effect for cannabinoids. Considering that COVID-19 mortality is largely attributed to disturbed immune regulation, the possible modulatory impact of cannabinoids in alleviating cytokine storms merits further investigation.

## 1 Introduction

Host-mediated inflammatory injury in the lung and associated blood vessels is a prominent feature of severe COVID-19 (Dorward et al., 2021). Recent genome-wide association studies (GWAS) uncovered at least two distinct genetic components underlying the severity of the disease: host susceptibility to viral infection and genetic propensity for adverse pulmonary inflammation (Pairo-Castineira et al., 2021). A growing body of evidence indicates that the immunological underpinning of severe COVID-19 is different from the moderate or mild forms, marked by differential response to immunosuppressive therapy among hospitalized patients. For example, administering glucocorticoids (i.e., dexamethasone) in hospitalized patients with respiratory failure is shown to substantially reduce the mortality rate, while the same treatment regimen among patients with less severe symptoms is generally harmful (The RECOVERY Collaborative Group, 2021).

A recent study investigated the relationship between substance use disorders (SUD) and the risk of severe COVID-19 in the United States and concluded that the risk of hospitalization and death due to COVID-19 is directly correlated with substance abuse, particularly with opioid use disorder (Wang Q. Q. et al., 2020). While fascinating, this observation may be biased due to comorbidities (such as hypertension, diabetes, cardiovascular disease (CVD), etc.) confounding the direct impact of SUD on sever COVID-19.

To objectively test whether drug abuse is causally related to an increased risk of COVID-19 adverse outcomes, we carried out Mendelian randomization (MR) analysis using summary statistics from high-powered GWAS of substance abuse (including cannabinoids (Johnson et al., 2020), opioids (Polimanti et al., 2020), alcohol (Sanchez-Roige et al., 2019)), medication-taking history (as a proxy trait for comorbidities such as CVD) (Wu et al., 2019), and the risk of COVID-19 hospitalization and respiratory failure (COVID-19 Host Genetic Initiative, release 4) (Supplementary Information S1). MR uses exposure-associated genetic variants as instrumental variables to investigate the causal relationship between exposure and the outcome (O’Connor and Price, 2018). Since genetic variants are randomly segregated at conception, MR resembles randomized controlled trials but is more robust to confounding than observational studies (Supplementary Information S1).

The motivation behind our analysis is to circumvent the confounding imparted by unmeasured comorbidities in the investigation of the causal relationship between SUD and COVID-19 adverse outcomes. The major limitation of Wang *et al.* study is that they could not control for comorbidities due to the cohort size limitation. Thus, although they reframed their null hypothesis to test whether SUD associated comorbidities contribute to patients’ risk to COVID-19 adverse outcomes, their observational analysis cannot assess the relationship between SUD and COVID-19: it is not clear whether the observed relationship is due to the higher prevalence of comorbidities among cases (compared to controls), or substance abuse has a real causal effect on the severity of COVID-19.

## 2 Methods

**FIGURE 1:**
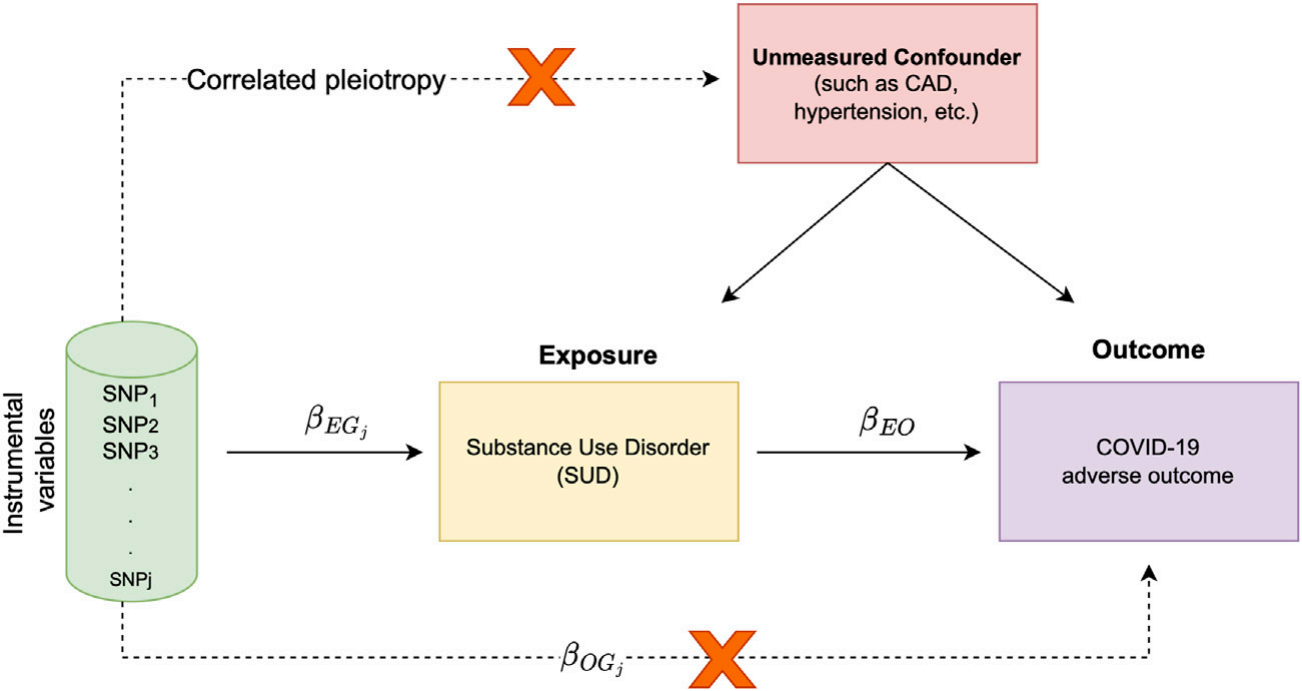
Schematic representation of study overview and assumptions underlying MR analysis; The target of inference in MR analysis is $\beta_{EO}$ (i.e., the causal effect of the exposure trait on the outcome). In this causal diagram, $\beta_{EG_{j}}$ is the measure of association between the genetic variant $G_{j}$ and the exposure trait (i.e., SUD in our analysis), $\beta_{OG_{j}}$ is the association of $G_{j}$ with the outcome (i.e., COVID-19 hospitalization or severe respiratory symptom) and $\beta_{EO}$ is the causal effect of exposure trait on the outcome. In our analysis, we make two important assumptions; 1) we assume that genetic variant $G_{j}$ affects both the exposure and outcome through the same causal pathway, and 2) our randomized instrument $G_{j}$ is not associated with any unmeasured confounder (i.e., there is no correlated pleiotropy).

The premise of MR relies on the association of exposure and outcome with genetic variants. Furthermore, MR can be simply carried out using only the GWAS summary statistics from the exposure and outcome traits. Since genetic variants are randomly segregated at conception, in MR analysis, genotypes are used as naturally occurring instruments. As such, a valid instrument is a variant associated with the exposure, but it is not associated with confounders of the exposure-outcome association. This instrumental variable is exclusively associated with the outcome *via* its effect on the exposure, revealing the causal relationship between the exposure and the outcome (Figure 1).

We explored the causal relationship between the SUD and COVID-19 adverse outcomes under four models: LCV, Egger regression, inverse-variance weighted linear regression (IVW) and weighted median (WM). A detailed description of MR methods is provided in Supplementary Information S1. For investigating the causal relationship in LCV, we used the R implementation of the model available at: https://github.com/lukejoconnor/LCV. For the remaining models, we used the TwoSampleMR (v.5.6) and MRPRESSO (Verbanck et al., 2018) packages in R.

### 2.1 Data source

We collected the summary statistics from published GWAS studies across multiple resources. For each trait analysed in our study, a brief description of the data source is provided below.

#### 2.1.1 COVID-19 host genetics initiative

We obtained GWAS summary statistics for “hospitalised COVID-19 cases vs. not hospitalised patients” and “very severe respiratory confirmed COVID-19 cases vs. population” from the meta-analysis round 4 of the COVID-19 host genetics initiative (https://www.covid19hg.org/about/). The COVID-19 host genetics initiative (HGI) is a collaborative project to identify genetic determinants of COVID-19 susceptibility, severity, and adverse outcomes. Results from the GWAS analysis are available to download freely from the project website.

The summary statistics for the hospitalisation record (B1_ALL) include meta-analysed GWAS weights across the 14,901,153 loci obtained from the analysis of 2,430 cases and 8,8478 controls with primarily European ancestry. The summary statistics for the severe respiratory symptom (A2_ALL) include meta-analysed GWAS weights across the 11,830,413 variants obtained from the analysis of 4,933 cases and 1,398,672 controls with primarily European ancestry.

#### 2.1.2 Psychiatric genomics consortium

We obtained the GWAS summary statistics for opioid dependence, alcohol dependence and cannabis use disorder from the Psychiatric Genomics Consortium (https://www.med.unc.edu/pgc/download-results/). These summary statistics are obtained from the relevant studies carried out under the PGC auspice and are briefly described below. Please see the cited papers for details pertaining to the analysis and GWAS results.

*Opioid use disorder (OUD).* The GWAS summary statistics for “opioid dependence” include GWAS weights across 5,986,961 loci obtained from the analysis of 4,503 opioid dependent, 4,173 opioid exposed and 32,500 opioid-unexposed controls (Polimanti et al., 2020).

*Alcohol use disorder (AUD).* We used the GWAS summary statistics for “alcohol use disorder” (Sanchez-Roige et al., 2019). The summary statistic includes meta-analysed GWAS weights across 16,213,999 loci obtained from the analysis 121,604 individuals in the UK biobank and 20,328 cases from the 23andMe. The authors used quantitative measures from the Alcohol Use Disorders Identification Test (AUDIT) to categorize participants as case and control.

*Cannabis use disorder (CUD).* The GWAS summary statistic for CUD include meta-analysed GWAS weights across 11,535,788 variants from the analysis of 20,916 cases and 363,116 controls from three cohorts including the Psychiatric Genomics Consortium Substance Use Disorders working group, iPSYCH, and deCODE (Johnson et al., 2020).

*UK Biobank medication-use.* We used GWAS summary statistics across six categories of medication use (Wu et al., 2019) (Supplementary Table S1). These summary statistics are obtained from the analysis of self-reported medication use across 23 medication categories among the participants of the UK Biobank.

## 3 Results

Consistent with the strong and significant impact of opioid use disorders (OUD) in Wang Q. Q. et al. analysis, we also identified a significant causality estimate for the opioid exposure trait (as a proxy for OUD) through the IVW regression method with a relatively similar effect size on both COVID-19 hospitalization and respiratory failure (hospitalization: OR_*IVW*_ = 5.23, *P*_*IVW*_ = 1.96E-11; severe respiratory symptoms: OR_*IVW*_ = 7.71, *P*_*IVW*_ = 1.25E-12). Since IVW causality estimates are sensitive to the presence of horizontal pleiotropy, we further tested whether opioids as a medication (prescribed in the clinical setting) exert any causal effect on COVID-19. To have a point of reference, we included medication classes that are primarily prescribed to treat high blood pressure and cardiovascular diseases (two suggested risk factors for severe COVID-19 (Hippisley-Cox et al., 2020)). We observed a significant effect of opioid medications on both COVID-19 hospitalization and respiratory failure (Table 1). Comparison of causality odds ratios suggests that opioid medications have a strong effect on severe respiratory symptoms (OR_*Egger*_ = 2.68, *P*_*Egger*_ = 8.01E-20) while only minimally (up to~3%) increase the odds of hospitalization.

**TABLE 1:** Summary of MR results for causal relationship between substance/medication traits (exposure) and COVID-19 adverse outcomes.

Substance/Medication category	COVID-19 outcome	LCV			Egger			IVW			WM		
		GCP	p-value	rg (SE)	p-value	OR (95%CI)		p-value	OR (95%CI)		p-value	OR (95%CI)	
Cannabis abuse	Hospitalized	**−0.82**	**8.07e–38**	0.14 (0.31)	**4.25e–03**	0.87 (0.87-0.88)	↓	**1.18e–08**	0.77 (0.77–0.78)	↓	0.74	0.97 (0.97–0.98)	
Cigarettes Per Day (Without UKB)		**−0.90**	**1.03e–30**	−0.27 (0.24)	**5.39e–31**	1.02 (1.02–1.03)	↑	**1.16e–122**	1.04 (1.04–1.05)	↑	**3.76e–12**	1.02 (1.02–1.03)	↑
Vasodilators used in cardiac diseases		**−0.73**	**9.05e–21**	0.17 (0.39)	**2.56e–16**	1.01 (1.00–1.01)	↑	**6.60e–28**	1.01 (1.01–1.02)	↑	**2.01e–16**	1.01 (1.01–1.02)	↑
Statin		0.05	0.03	0.24 (0.26)	**3.46e–56**	1.06 (1.05–1.07)	↑	**8.32e–199**	1.08 (1.07–1.08)	↑	**2.31e–62**	1.09 (1.08–1.10)	↑
Opioid (medication)		0.00	0.94	0.40 (0.38)	**9.20e–48**	1.03 (1.03–1.04)	↑	**2.06e–56**	1.02 (1.02–1.03)	↑	**4.98e–42**	1.03 (1.03–1.04)	↑
Opioid exposure		0.00	0.89	−0.98 (1.34)	1.21e–01	0.50 (0.21–1.20)		**1.96e–11**	5.23 (3.23–8.49)	↑	0.40	1.24 (0.75–2.04)	
Diuretics	Severe Respiratory	−0.53	**1.21e–06**	−0.31 (0.20)	0.15	1.22 (0.93–1.59)		**2.66e–15**	0.47 (0.39–0.57)	↓	0.18	0.87 (0.70–1.07)	
Antithrombotic agents		0.22	**4.64e–4**	−0.39 (0.52)	0.70	1.07 (0.75–1.54)		0.016	0.74 (0.58–0.95)		0.01	1.38 (1.07–1.77)	
Cannabis abuse		−0.11	9.80e–3	0.95 (0.28)	0.84	1.01 (0.91–1.12)		0.36	1.04 (0.95–1.14)		0.02	0.87 (0.77–0.98)	
Agents acting on the renin-angiotensin system		**−0.62**	0.01	−0.43 (0.18)	0.20	0.80 (0.56–1.13)		**1.87e–17**	0.36 (0.29–0.46)	↓	0.01	0.70 (0.53–0.92)	
Alcohol intake frequency		**−0.60**	0.03	0.42 (0.17)	0.19	0.70 (0.41–1.19)		**8.92e–4**	0.53 (0.37–0.77)	↓	**2.08e–07**	0.30 (0.19–0.48)	↓
Opioid (medication)		−0.08	0.59	0.12 (0.30)	**8.01e–20**	2.68 (2.17–3.31)	↑	**1.50e-16**	1.85 (1.60–2.14)	↑	**6.11e–07**	1.43 (1.24–1.65)	↑
Opioid exposure		−0.01	0.78	*1.72 (10.63)	0.12	0.50 (0.21–1.20)		**1.25e–12**	7.71 (4.39–13.55)	↑	0.41	1.24 (0.74–2.07)	

Note: COVID-19 adverse outcomes include both hospitalization and severe respiratory symptoms. Significant values are highlighted in bold, and arrows indicate the direction of causality. According to O’Connor and Price (2018) the genetic causality proportion (GCP) greater than |0.6| is suggestive of a causal relationship. Negative GCP, values are indicative of a reverse causality relationship. The null hypothesis for the reported p-value in LCV analysis is GCP = 0. For each category of COVID-19 outcome, only exposure traits that reached the significance level across at least one MR method are shown. rg, The estimate of genetic correlation from the LCV method; Egger, Egger regression; IVW, inverse-variance weighted linear regression; WM, Weighted median. (* LD score regression estimate of rg is not bounded. Estimates outside of [−1, 1] are possible; however, the extremely large standard error indicates that estimates of genetic correlation, in this case, is unstable.)

Our study showed that two classes of medications, including vasodilators and statin (used for cardiac diseases) are causally related to the risk of COVID-19 hospitalization (Table 1). The impact of vasodilators on the risk of hospitalization was almost negligible (up to 1% increase), but statin showed a stronger effect on the risk of hospitalization (OR_*Egger*_ = 1.06, *P*_*Egger*_ = 3.46E-56). The positive genetic correlation of both medication classes with COVID-19 hospitalization (vasodilators: *r*_g_ = 0.17, statin: *r*_g_ = 0.24) confirms the previously reported higher baseline prevalence cardiovascular conditions among hospitalized patients (Wang B. et al., 2020). Neither of the two classes of medication showed a significant causality relationship with COVID-19 respiratory failure. We also detected a significant negative causal effect for diuretics and medication class acting on the renin-angiotensin system (RAAS) with the risk of COVID-19 reparatory failure. Since both medication classes are primarily prescribed for patients with hypertensive disease, this negative causal effect further underlines the importance of ACE inhibitors in controlling COVID-19 severe illness (diuretics: OR_*IVW*_ = 0.47, P_*IVW*_ = 2.66E-15; RAAS: OR_*IVW*_ = 0.36, *P*_*IVW*_ = 1.87E-17).

We detected evidence for a (negative) causal effect of cannabis abuse with COVID-19 hospitalization (Hospitalization: OR_*IVW*_ = 0.77, *P*_*IVW*_ = 1.18E-08, Table 1). There is no clear mechanistic evidence linking cannabis use disorder (CUD) to COVID-19 symptoms, but we hypothesize that this negative causal effect is exerted through immunomodulatory pathways related to cannabinoid receptors. We found this observation interesting since Wang et al. also observed a protective association for lifetime CUD and COVID-19 (OR = 0.85, *p* = 0.006).

## 4 Discussion

Inspired by the recent analysis of SUD and COVID-19 outcomes from electric health records in the United States (Wang Q. Q. et al., 2020), we investigated the causality of SUD on COVID-19 adverse outcomes while rigorously controlling for the plausible confounding by comorbidities in the association between SUD with COVID-19 hospitalization and death. We utilized GWAS summary statistics across four classes of SUD—i.e., opioid use disorder (OUD), cannabis use disorder (CUD), alcohol use disorder (AUD)—and medication taking history (as a proxy trait for comorbidities) in an MR framework to investigate the causality of SUD on COVID-19 adverse outcomes. We replicated the reported causal relationship between opioid exposure trait (as a proxy for OUD) with COVID-19 hospitalization and respiratory failure. However, contrary to Wang et al., we identified a negative causal relationship between cannabis abuse and COVID-19 hospitalization (OR_*IVW*_ = 0.77, *P*_*IVW*_ = 1.18E-08). We believe this result is noteworthy as the earlier report could not control for SUD-associated comorbidities such as type 2 diabetes, hypertension, and diseases of heart, kidney, lung, and liver that substantially contribute to COVID-19 adverse outcomes (Richardson et al., 2020; Rosenthal et al., 2020).

In line with our finding, a recent retrospective analysis of 1,831 COVID-19 inpatient admissions across two UCLA hospitals identified that, while controlling for age, body mass index (BMI), sex, race, tobacco smoking history, and SUD-associated comorbidities, active cannabinoid use is associated with shorter hospitalization, lower ICU admission, and less need for mechanical ventilation (Shover et al., 2022). Notably, immunomodulatory markers—including C-reactive protein (CRP), ferritin, D-dimer, and procalcitonin—were significantly lower among the patients with an active cannabinoid taking history. Considering that progression to severe COVID-19 is attributed primarily to the hyperinflammatory state where the level of pro-inflammatory markers (i.e., CRP, ferritin and serum cytokines such as IL-1B, IL-8 and sTNFR) is elevated, it is likely that the favorable effect of cannabinoids in mitigating adverse outcomes is mediated through dampening the immunomodulatory response.

It is important to note that in our MR framework, the total causal effect is essentially the change in COVID-19 outcome resulting from intervening in SUD. By definition, this total effect comprises the direct effect and mediating effect (Burgess et al., 2017). In the absence of a clear mechanistic pathway linking cannabis use disorder (CUD) to COVID-19 symptoms, we hypothesize that the causal evidence we identified for CUD is largely derived through a mediator trait related to immunomodulatory response. Dissecting the direct (non-mediated) effect of CUD on COVID-19 adverse outcomes from the total effect requires a comprehensive assessment of the plausible mediator trait(s) (e.g., CRP) and availability of sufficiently powered GWAS, which is beyond the scope of our analysis.

Our results also show a significant effect of diuretics and angiotensin-converting enzyme (ACE) inhibitors on reducing severe respiratory failure among COVID-19 patients (Table 1). The administration of these drugs in COVID-19 patients has been the subject of major discussion and stipulations, especially during the early days of the pandemic (Guzik et al., 2020). Our results not only reaffirm the benefits of these drugs in mitigating COVID-19 adverse outcomes among patients with cardiovascular comorbidities (Hippisley-Cox et al., 2020; Rosenthal et al., 2020) but also demonstrate how the MR framework can be rapidly utilized to derive reliable clinical recommendations in the event of a global health emergency.

A major limitation of our analysis is that we could not stratify the clinical outcomes by the major SARS-CoV-2 variants. It is now evident that the delta variant of SARS-CoV-2 (B.1.617.2) was associated with more adverse outcomes compared to the alpha variant (Twohig et al., 2022). Although we used GWAS summary statistics from COVID-19 Host Genetic Initiative (release 4, October 20, 2020) from around the time when delta (B.1.617.2) was the predominant lineage in the US and Europe, the extent to which the subvariants class confounds the designation of adverse outcomes in patients is not clear to us. Nevertheless, supposing that the host’s susceptibility to infection remains mostly independent of the virus subvariant, at least up until the emergence of a more transmissible Omicron strain (BA.1), we believe the causal association that we identified is unaffected by this confounding.

In conclusion, our MR analysis confirms the causal relationship between opioids and severe COVID-19 illness. However, our MR analysis questions the validity of the causal relationship between CUD and COVID-19 severe illness. A recent study showed that treatment with cannabis compounds significantly reduces cytokine secretion in lung epithelial cells and, therefore, may be useful in alleviating severe symptoms in COVID-19 patients (Anil et al., 2021). The fact that a great deal of COVID-19 mortality is attributed to immune dysregulation and cytokine storm, the possible modulatory impact of cannabinoids merits further investigation. Besides, it is shown that cannabidiol (CBD) blocks viral replication in lung epithelial cells through the up-regulation of endoplasmic reticulum (ER) stress response and interferon signaling pathways. Intriguingly, medical history of oral CBD use was associated with a reduced COVID-19 test-positivity rate (Nguyen et al., 2022). It is important to note that our finding explicitly do not encourage cannabis use. Our analysis does not provide insight into the physiological mechanism linking COVID-19 adverse outcomes with cannabinoid use, nor did we intend to promote cannabis use as an alternative therapy for alleviating COVID-19 symptoms. Cannabis abuse is shown to be significantly associated with an increased risk of psychiatric disorders (Hill et al., 2017) and delineation of any protective effect against COVID-19 adverse outcomes requires rigorous biochemical follow-up that is beyond the scope of our statistical analysis.

## Data Availability

Publicly available datasets were analyzed in this study. This data can be found here: https://www.covid19hg.org/results/r4/ (COVID-19 Host Genetics Initiative), https://pgc.unc.edu/for-researchers/download-results/ [Psychiatric Genomics Consortium (PGC)], https://cnsgenomics.com/content/data/ (UK Biobank medication-taking GWAS), https://github.com/zdz-lab/COVID19_MR/.
