# Supplementary material for: Substance abuse and the risk of severe COVID-19: Mendelian randomization confirms the causal role of opioids but hints a negative causal effect for cannabinoids
Source: Front Genet. 2022 Dec 13;13:1070428. doi: 10.3389/fgene.2022.1070428 (PMC9792508; doi:10.3389/fgene.2022.1070428)
Supplement: Supplementary file 1 [file DataSheet1.docx]

**Supplementary Information**

Substance abuse and the risk of severe COVID-19: Mendelian randomization confirms the causal role of opioids but hints a negative causal effect for cannabinoids.

Jabalameli and Zhang

**Supplementary note**

To infer the causal relationship between SUD and COVID-19 adverse outcomes (Hospitalization and severe respiratory symptom), we conducted a Mendelian Randomization (MR) analysis. Unlike randomized trials that are costly, time prohibitive and, in the case of SUD, unethical to carry out, MR offers an amenable alternative for inferring causal relationships in a timely manner and efficiently. Furthermore, the application of MR in inferring causality also circumvents the biased conclusions occasionally associated with observational studies due to confounding and reverse causality (**Figure S1**).

**Figure S1:** Schematic representation of assumptions underlying MR analysis.


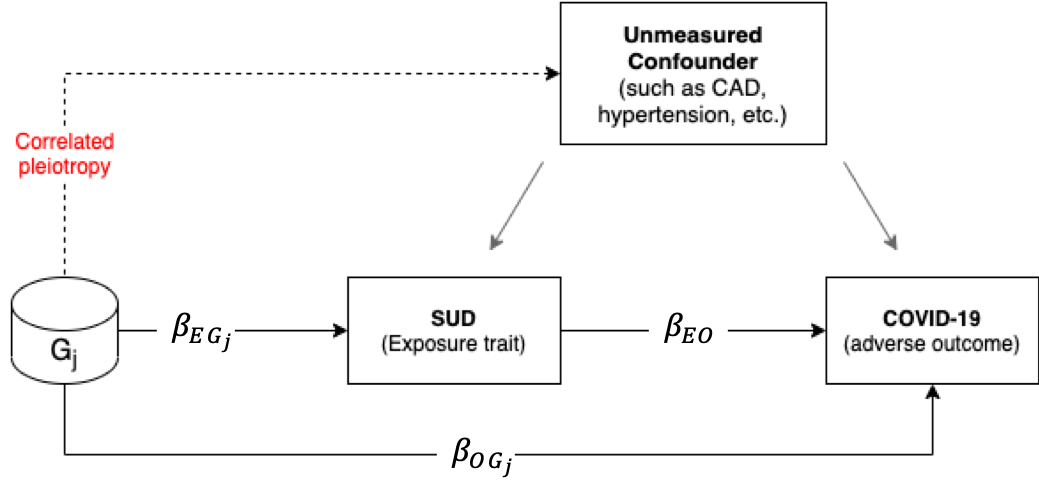


The target of inference in MR analysis is $\beta_{EO}$ (i.e. the causal effect of the exposure trait on the outcome). To circumvent the confounding effect of unmeasured traits (such as cardiovascular disorders and hypertension) that may be correlated with both the exposure and outcome (identified by grey arrows), we use genetic variants associated with exposure but are not associated with outcome, except through their effect on exposure. In this causal diagram, $\beta_{EG_{j}}$ is the measure of association between the genetic variant $G_{j}$ and the exposure trait (SUD in our analysis), $\beta_{OG_{j}}$ is the association of $G_{j}$ with the outcome (i.e. COVID-19 hospitalization or severe respiratory symptom) and $\beta_{EO}$ is the causal effect of exposure trait on the outcome. In our analysis, we make two important assumptions; 1) we assume that genetic variant $G_{j}$ affects both the exposure and outcome through the same causal pathway, and 2) our randomized instrument $G_{j}$ is not associated with any unmeasured confounder (i.e. there is no correlated pleiotropy).

**Mendelian Randomization methods**

In an MR analysis, in principle, we derive the causal effect (for any one SNP) as the ratio of the SNP-outcome association to the SNP-exposure association. Specifically, the hypothesized relationship between the exposure and outcome for each instrumental variable j (out of J) is given by:

|  | $\beta_{{OG}_{j}}= \beta_{OE} \beta_{{EG}_{j}}$ | (1) |
| --- | --- | --- |

where $\beta_{{OG}_{j}}$is the effect size of the instrumental variable (${SNP}_{j}$) association with the outcome (in the case of our analysis, covid-19 hospitalization or severe respiratory outcome), $\beta_{{EG}_{j}}$ is the effect size of the association between the instrumental variable (${SNP}_{j}$) and the exposure (for example OUD or CUD in our analysis), and $\beta_{OE}$ is the causal effect of E (exposure) on O (outcome). In GWAS summary statistics, all variants have been coded to reflect the positive SNP-exposure association; hence $\beta_{{OG}_{j}}$ is always non-zero. Derivation of causal effect $\beta_{OE}$ motivates the use of ratio so that for each instrumental variable j, the causal effect can be estimated by:

|  | $\beta_{{OE}_{j}}= {\beta_{{OG}_{j}}}/{\beta_{{EG}_{j}}}$ | (2) |
| --- | --- | --- |

The WM (or IVW) method estimates the causal effect simply as a variance weighted (or an inverse variance-weighted) average of all instrumental variable ratios ($\beta_{{OE}_{j1}}, \beta_{{OE}_{j2}}, \beta_{{OE}_{j3}}, \ldots;j \in J$). The Egger method extends this basic principle of MR to allow all variants to exert a pleiotropic effect on the outcome via the regression model based on the following:

|  | $\beta_{{OG}_{j}}= {\alpha_{j}+ \beta}_{OE} \beta_{{EG}_{j}}$ | (3) |
| --- | --- | --- |

where $\alpha_{j}$ is the pleiotropic effect of instrumental variable SNP $j$.

The LCV method principally follows a similar framework as the Egger regression, but it is more mathematically involved and relies on the estimation of the Latent Causal Variable (LCV) that mediates the genetic correlation (r_g_) between the E and O traits. Under the LCV model, the relative proportion of heritability (between the exposure and outcome) that is attributable to the shared factor is calculated. To quantify the magnitude of heritability explained by the shared factor, O’Connor and Pric ^1^, introduce the “genetic causality proportion (GCP)” that ranges from -1 to 1 depending on the direction of causality. Under this model, a fully genetically causal relationship (defined as $\hat{|GCP|}=1$) is inferred when the entire effect of the exposure trait on the outcome is derived from the genetic component underlying the shared factor ($\beta_{OE}$= $\sum\beta_{{OE}_{j}})$. The LCV method computes the significance of GCP against the null hypothesis where $\hat{GCP}=0.$ According to O’Connor and Pric ^1^, GCP estimates larger than 0.6 ($\hat{|GCP|}>0.6$) suggest a plausible causal relationship.

**Table S1:** Summary of medication-use GWAS.

| Data Source | Trait | No. of cases | No. of controls | No. of total tested GWAS variants | No. independent GWAS hit (P ≤ 5E-8) | Reference |
| --- | --- | --- | --- | --- | --- | --- |
| HGI | Hospitalised COVID-19 cases vs. not hospitalised | 2,430 | 8,8478 | 14,901,153 | 14 | [www.covid19hg.org](http://www.covid19hg.org) |
|  | Very severe respiratory confirmed COVID-19 cases vs. population | 4,933 | 1,398,672 | 11,830,413 | 542 |  |
| PGC | Opioid use disorder (OUD) | 8,676 | 32,500 | 5,986,961 | 20^♠^ | Polimanti *et al.*^4^ |
|  | Alcohol use disorder (AUD)^♣^ | 121,604 UK Biobank  20,328 23&Me | | 16,213,999 | 83 | Sanchez-Roige *et al.*^5^ |
|  | Cannabis use disorder (CUD) | 20,916 | 363,116 | 11,535,788 | 752 | Johnson *et al.*^6^. |
| UK Biobank | Agents acting on the renin-angiotensin system | 62,752 | 174,778 | 7,288,504 | 184 | Wu et al.^7^ |
|  | Antithrombotic agents | 67,653 | 85,986 |  | 13 |  |
|  | Diuretics | 34453 | 194633 |  | 102 |  |
|  | Opioid (medication) | 22982 | 55826 |  | 3 |  |
|  | Statin | 73475 | 216910 |  | 97 |  |
|  | Vasodilators used in cardiac diseases | 5546 | 237113 |  | 3 |  |

♣ In Sanchez-Roige *et al.,* AUD is treated as a quantitative trait. The authors calculated the Alcohol Use Disorder Identification Test (AUDIT) score across all participants and used different thresholds to dichotomize the cohort into alcohol consumption (**AUDIT-C**, N = 121,604) and alcohol problem (**AUDIT-P**, N = 121,604) sub-cohorts.

♠ Given the comparatively lower number of total variants tested for OUD, we used the lenient p-value threshold p ≤ 5E-6 to select instrumental variables.

**References**

1. O’Connor, L. J. & Price, A. L. Distinguishing genetic correlation from causation across 52 diseases and complex traits. *Nat. Genet.* (2018). doi:10.1038/s41588-018-0255-0

2. Bowden, J., Smith, G. D. & Burgess, S. Mendelian randomization with invalid instruments: Effect estimation and bias detection through Egger regression. *Int. J. Epidemiol.* (2015). doi:10.1093/ije/dyv080

3. Verbanck, M., Chen, C. Y., Neale, B. & Do, R. Detection of widespread horizontal pleiotropy in causal relationships inferred from Mendelian randomization between complex traits and diseases. *Nat. Genet.* **50**, (2018).

4. Polimanti, R. *et al.* Leveraging genome-wide data to investigate differences between opioid use vs. opioid dependence in 41,176 individuals from the Psychiatric Genomics Consortium. *Mol. Psychiatry* (2020). doi:10.1038/s41380-020-0677-9

5. Sanchez-Roige, S. *et al.* Genome-wide association study meta-analysis of the alcohol use disorders identification test (AUDIT) in two population-based cohorts. *Am. J. Psychiatry* **176**, (2019).

6. Johnson, E. C. *et al.* A large-scale genome-wide association study meta-analysis of cannabis use disorder. *The Lancet Psychiatry* (2020). doi:10.1016/S2215-0366(20)30339-4

7. Wu, Y. *et al.* Genome-wide association study of medication-use and associated disease in the UK Biobank. *Nat. Commun.* (2019). doi:10.1038/s41467-019-09572-5
